# Cytomegalovirus-Associated Colitis as a Cause of Lower Gastrointestinal Bleeding in Kidney Transplant Recipients: A Single-Centered Study

**DOI:** 10.7759/cureus.62422

**Published:** 2024-06-15

**Authors:** Arz Muhammad, Raja Taha Yaseen Khan, Tajammul Waqar, Ali Hyder, Sidra German, Syed Mudassir Laeeq, Zain Majid, Abbas Ali Tasneem, Abdullah Nasir, Nasir Hassan Luck

**Affiliations:** 1 Hepatogastroenterology, Sindh Institute of Urology and Transplantation, Karachi, PAK; 2 Nephrology, Sindh Institute of Urology and Transplantation, Karachi, PAK; 3 Gastroenterology, Chandka Medical College, Shaheed Mohtarma Benazir Bhutto Medical University, Larkana, PAK; 4 Department of Medicine, Jinnah Medical and Dental College, Karachi, PAK

**Keywords:** colitis, c.diff colitis, frequency, renal transplantation, lower gi bleeding, cytomegalovirus (cmv)

## Abstract

Introduction

Cytomegalovirus (CMV) is the most common viral pathogen affecting patients undergoing solid organ transplantation. It is often the most important infection for patients who have undergone kidney transplantation. Clinical presentations of cytomegalovirus infection range from asymptomatic infection to organ-specific involvement. This study aimed to determine the frequency of cytomegalovirus-associated colitis in kidney transplant recipients (KTRs) presenting with lower gastrointestinal bleeding.

Methods

After the approval of the ethical review committee of the Sindh Institute of Urology and Transplantation (ERC-SIUT), this cross-sectional study was conducted at the Department of Hepatogastroenterology at the Sindh Institute of Urology and Transplantation from January 2021 to December 2021. All the KTRs (six months after the transplantation) of either gender and aged between 18 and 65 years, presenting with lower gastrointestinal (GI) bleeding as per the operational definition, were enrolled in the study. Those patients who were either unfit for the endoscopy or refused to give consent were excluded from the study. Colonic biopsies were reviewed by a consultant histopathologist for the features of CMV infection.

Results

A total of 95 renal transplant recipients of either gender or age above 18 to 65 years with lower GI bleeding were included in the study. Among them, 84 (88.4%) were males, while 11 (11.6%) were females. The mean age of the patients included in the study was 37±11 years. The most common presenting complaint was fresh bleeding per rectum, which was observed in 73 (76.8%). The most common findings observed on colonoscopy in KTRs with bleeding per rectum were colonic ulcers and erosions noted in 41 (43.1%) and 36 (37.3%) patients, respectively. On histopathology, CMV colitis was noted in 21 (22.1%) patients. On comparison of different baseline variables, the presence of fresh bleeding per rectum and the presence of both ulcers and erosions on colonoscopy were the factors significantly associated with CMV colitis in KTRs.

Conclusion

CMV colitis is a prevalent condition in KTRs, presenting with lower GI bleeding. Despite the significant occurrence, the levels of CMV viremia were not associated with CMV colitis, suggesting that diagnosis should rely on histopathological confirmation. Prophylaxis during periods of high immunosuppression is crucial to reducing the incidence of CMV infections and improving both graft function and patient survival.

## Introduction

Renal transplantation is the treatment of choice for chronic kidney disease. After renal transplantation, with the subsequent use of immunosuppressive medications, a variety of immunologic, infectious, and surgical complications can occur [1]. Gastrointestinal complications in kidney transplant recipients (KTRs) include oral lesions, esophagitis, peptic ulcers, diarrhea, colonic disorders, and malignancy [2].

Colonic disorders are not only limited to opportunistic infections like cytomegalovirus (CMV) but also include diverticulitis, intestinal ischemia, Epstein-Barr virus (EBV)-associated lymphoproliferative disorders, and colorectal and anal carcinomas [3]. CMV usually involves the colon, mostly the sigmoid and rectum [3]. Wadhwa et al. reported 15.5% of CMV colitis in KTRs, presenting with a variety of symptoms (diarrhea, weight loss, bleeding per rectum, etc.) [4]. D. Bhadauria reported a prevalence of 3% of CMV colitis, but the author didn’t specify the presenting symptoms in his retrospective study [5]. Lempinen M. and his colleagues evaluated the colonic biopsies for CMV infection and reported a 21% prevalence in patients with endoscopic findings of CMV colitis amongst the KTRs [6].

Although CMV colitis often presents with large bowel-type diarrhea, it may also present with overt or obscure GI bleeding. The bleeding from the GI tract is a treacherous manifestation of CMV colitis and may result in significant blood loss, leading to hemodynamic changes, acute renal dysfunction, and overall health deterioration. Even patients may present with only mild abdominal symptoms like mild abdominal pain or mild diarrhea associated with iron deficiency anemia [7].

The frequency of CMV-associated colitis in post-renal transplant settings has been reported to be as high as 15.5% [4]. The index of suspicion should, therefore, be kept high for CMV-associated lower GI bleed in these patients who are maintained on immunosuppressive agents (steroids, calcineurin inhibitors, mammalian targets of rapamycin inhibitors, and inosine monophosphate dehydrogenase inhibitors) to prevent graft rejection. These immunosuppressive medications, however, not only predispose the patient to infections like CMV and its complications, including colitis-related GI bleeds but also affect both the graft and the patient's survival [8].

The high variability in reported prevalence rates, ranging from 3% to 21%, underscores the necessity for more localized research to understand the true burden of CMV colitis in different populations [5,6]. Furthermore, the complications associated with CMV colitis, including significant blood loss, hemodynamic changes, acute renal dysfunction, and overall health deterioration, necessitate a high index of suspicion for timely diagnosis and intervention.

Given the potential severity of CMV colitis and its impact on patient and graft survival, understanding its prevalence and associated factors in the local population is crucial. Therefore, this study aims to address this gap by determining the frequency of CMV-associated lower GI bleeding in kidney transplant recipients at a single center. 

By elucidating the prevalence and clinical characteristics of CMV colitis in this specific patient population, the study aims to contribute to improved clinical outcomes through enhanced awareness, early diagnosis, and effective management of this condition.

## Materials and methods

After the approval of the ethical review committee of the Sindh Institute of Urology and Transplantation (ERC-SIUT), this cross-sectional study was conducted at the Department of Hepatogastroenterology at the Sindh Institute of Urology and Transplantation from January 2021 to December 2021. All the KTRs (six months after the transplantation) of either gender and aged between 18 and 65 years, presenting with overt lower gastrointestinal (GI) bleeding as per the operational definition, were enrolled in the study (Table 1). Those patients who were either unfit for the endoscopy or refused to give consent were excluded from the study.

**Table 1:** Operational definition of CMV colitis and overt lower GI bleeding CMV: Cytomegalovirus; GI: Gastrointestinal

Operational Definitions:
CMV Colitis [6,7]: CMV colitis was diagnosed on a colonic mucosal biopsy, revealing the typical nuclear inclusion bodies with an “owl eye appearance.”
Overt lower GI bleeding [8,9]: Overt lower GI bleeding was defined as the passage of fresh per rectal bleeding or the passage of black tarry stool.

Sampling technique and sample size

Patients were enrolled using the technique of non-probability consecutive sampling. Based on previous estimates, CMV colitis was observed in 15.5% of patients [5]. Taking a margin of error of 7.5% and a confidence level of 95%, a total of 90 patients were needed for this study.

Data collection procedure

After the approval of this study from the ERC and SIUT, all RTRs, after six months of the kidney transplant presenting with lower GI bleeding, were enrolled in the study. Renal transplant recipients fulfilling the inclusion criteria then underwent colonoscopy after informed consent.

A colonoscopy was performed by an expert endoscopist using the video endoscope Olympus GIF-XP190, and findings like ulcers, erosion, or polys were noted.

Colonic biopsies were taken at least twice each from the sigmoid colon, descending colon, transverse colon, ascending colon, caecum, and ileocecal valve. All biopsy specimens were immersed in formalin and then embedded in paraffin. Biopsy slides were reviewed by a consultant histopathologist with a minimum of three years of experience, and the presence of giant cells with pleomorphic nuclei containing basophilic inclusions (owl’s eyes, halo rim) was labeled as CMV colitis. Post-procedure, all the patients were observed for any possible complications like bleeding, abdominal pain, or perforation. All tests were done free of charge, as per institutional policy.

All information, along with age, gender, symptoms (abdominal pain, diarrhea, passage of black stool, or melena), and etiology of bleeding, was noted by the researcher.

Data analysis

IBM Corp. Released 2013. IBM SPSS Statistics for Windows, Version 22.0. Armonk, NY: IBM Corp. was used for data entry and analysis. Frequencies and percentages were computed for categorical variables like gender, etiology, and lower GI bleeding. Quantitative values like age and duration of bleeding were presented as mean ± standard deviation. Effect modifiers like age, gender, and duration of bleeding were controlled through stratification. A post-stratification Chi-square test was applied, and p values < 0.05 were considered statistically significant.

## Results

A total of 95 renal transplant recipients of either gender or age above 18 to 65 years with lower GI bleeding were included in the study. Among them, 84 (88.4%) were males, while 11 (11.6%) were females. The mean age of the patients included in the study was 37±11 years. Most of the patients (56 [53%]) had an age > 35 years, while 42 (47%) had an age < 35 years. Hypertension was the most common comorbidity observed in 57 (60%) patients, followed by asthma (27 [28%]), diabetes (16 [17%]), and ischemic heart disease (7 [7.4%]). The most common presenting complaint was fresh bleeding per rectum, which was observed in 73 (76.8%), followed by Malena in 22 (23.2%) patients, respectively (Table 2).

**Table 2:** Baseline characteristics of the population included in the study (n: 95) KTRs: Kidney transplant recipients; CMV: Cytomegalovirus; TLC: Total leucocyte count; INR: International normalized ratio

Study population		n (%)
Gender	Male	84 (88.4)
	Female	11 (11.6)
Age(years)	≥35	53 (56)
	<35	42 (44)
Symptoms	Fresh bleeding per rectum	73 (76.8)
	Malena	22 (23.2)
Comorbidities	Hypertension	57 (60)
	Diabetes	16 (17)
	Asthma	27 (28)
	Ischemic Heart Disease	7 (7.4)
Colonoscopic Finding	Erosions	36 (37.9)
	Ulcers	41 (43.1)
	Polypoidal Growth	7 (7.4)
	Pseudomembranes	5 (5.3)
	Normal	6 (6.3)
Duration of lower GI bleed among KTRs	≤ 1 week	77 (81.1)
	>1 week	18 (18.9)
CMV antigenimia	≥25000	11 (11.2)
	<25000	84 (88.4)
CMV colitis on Histology	Present	21 (22.1)
	Absent	74 (77.9)
Mean age (years± S.D)		37±11
Hemoglobin(g/dL)		9.58±2.11
TLC(x10^9^/L)		8.9±5
Platelet Count(x10^9^/L)		154±61
INR		1.05±0.07
Serum Creatinine(mg/dl)		1.7±1.0

The duration of bleeding per rectum was less than one week in most of the patients (77 [81.1%]). The most common findings observed on colonoscopy in KTRs with bleeding per rectum were colonic ulcers and erosions noted in 41 (43.1%) and 36 (379.9%) patients, followed by polypoidal growth and pseudomembranes in seven (7.4%) and five (5.3%), respectively.

Low levels of viremia, i.e., CMV DNA levels of <25,000 copies/ml, were noted in 84 [88%] KTRs with bleeding per rectum, while 11 (11.2%) patients had high levels of viremia with CMV DNA levels ≥25000. On histopathology, CMV colitis was noted in 21 (22.1%) patients (Table 2). On comparison of different baseline variables, the presence of fresh bleeding per rectum and the presence of both ulcers and erosions on colonoscopy were the factors significantly associated with CMV colitis in KTRs (Table 3).

**Table 3:** Comparison of baseline variables in terms of the presence of CMV colitis CMV: Cytomegalovirus; IHD: Ischemic heart disease; TLC: Total leucocyte count; INR: International normalized ratio

Variable		CMV colitis		p-value
		Present (n-21) N (%)	Absent (n-74) N (%)	
Gender	Males	12(57.1)	72 (97.3)	0.929
	Females	9 (42.9)	2(2.7)	
Age (years)	<=36 Years	10 (47.6)	38 (51.4)	0.763
	>36 Years	11 (52.4)	36 (48.6)	
Hypertension	Yes	15 (71.4)	42 (56.8)	0.226
	No	6 (28.6)	32 (43.2)	
Diabetes	Yes	1 (4.8)	15 (20.3)	0.048
	No	20 (95.2)	59 (79.7)	
Asthma	Yes	4 (19)	23 (31.1)	0.281
	No	17 (81)	51 (68.9)	
IHD	Yes	0 (0)	7 (9.5)	0.143
	No	21 (100)	67 (90.5)	
Malena	Yes	4 (19)	18 (24.3)	0.613
	No	17 (81)	56 (75.7)	
Fresh Bleeding Per Rectum	Yes	15 (71.4)	58 (79.4)	0.039
	No	6 (28.6)	40 (54.1)	
Hemoglobin(g/dl)		9.45±1.89	9.74 ±2.52	0.453
TLC (x 10^9^/L)		9.0±4.7	8.8±3.9	0.086
Platelets (x 10^9^/L)		156±59	151±62	0.259
INR		1.1±0.64	1.14±0.58	0.326
Serum Creatinine (mg/dl)		1.83±0.94	1.68±0.64	0.147
Colonoscopic Findings	Ulcers	18 (4.8)	23(31.1)	<0.001
	Pseudomembranous	3 (14.3)	2 (2.7)	
	Normal mucosa	2 (9.5)	33 (44.6)	
	polypoidal growth	2 (9.5)	5 (6.8)	
	Erosions	13 (0)	23 (31.1)	

## Discussion

Renal transplantation is the treatment of choice for end-stage renal disease patients. After transplantation, there is an increased risk of various gastrointestinal tract (GI) disorders. The incidence of GI problems varies in renal transplant recipients (RTRs) from 20% to 55 [2,4]. Cytomegalovirus (CMV) is the most common viral infection contracted after kidney transplantation. However, its clinical presentation ranges from an asymptomatic infection to organ-specific involvement [10]. Most symptomatic infections manifest as fever, fatigue, and cytopenia. The gastrointestinal tract is the most common site of tissue-invasive CMV infection, with abdominal pain and diarrhea as the most prominent symptoms. In severe cases, gastrointestinal hemorrhage and perforation occur. CMV infection can involve the liver, lungs, heart, pancreas, and kidneys. Chorioretinitis, which is a common CMV manifestation in HIV-infected patients, is very rare in solid-organ recipients [10,11]. CMV disease usually presents with fever, diarrhea, and abdominal pain. Nausea and impaired gastric emptying may reflect upper GI involvement [2,12]. In severe CMV gastroenteritis or colitis, GI bleeding and, on some occasions, even perforation may occur [13]. 

CMV usually involves the colon, mostly the sigmoid and rectum [3]. Wadhwa et al. reported a prevalence of 15.5% of CMV colitis in KTRs presenting with a variety of symptoms (diarrhea, weight loss, bleeding per rectum, etc.) [4]. On the other hand, D. Bhadauria reported a prevalence of 3% of CMV colitis, but the author didn’t specify the presenting symptoms in his retrospective study [5]. Lempinen M et al. evaluated the colonic biopsies for CMV infection and reported a 21% prevalence in patients with endoscopic findings of CMV colitis amongst the KTRs [6]. Other studies reported the prevalence of CMV infection at 19% and 16.6% in KTRs [14,15].

CMV prophylaxis plays an important role in the prevalence of the infection in post-transplant settings. CMV infections can develop in 10% to 60% of kidney transplant patients without prophylaxis and treatment. It occurs early, during the phase of the highest immunosuppressive load [16].

In our study, we observed that CMV-associated colitis presenting with lower gastrointestinal bleeding amongst renal transplant patients was present in 21 (22.1%) patients, which is much lower than in other studies. However, it is quite low in developed countries like Europe and the USA. But some studies also had a higher prevalence than our study (14% to 38%) [17]. The reason for this difference was due to the serologic incompatibility of CMV, which leads to the highest risk of CMV disease [18]. In our study, there was no effect of the type of immunosuppressive regimen on the CMV prevalence.

In our study, we observed that CMV infection was not associated with any comorbidities, which was a finding comparable to the previous studies done in the KTRs [14]. Similar to the previous studies, we observed that the presence of ulcers and erosions on colonoscopy was significantly associated with the presence of CMV colitis in our KTRs [4].

To the best of our knowledge, this is the first study estimating the prevalence of CMV as a cause of lower GI bleeding in KTRs in our population.

However, certain limitations can be attributed to our study. At first, this study included a relatively small cohort of 95 renal transplant recipients, which may limit the generalizability of the findings to the broader population of kidney transplant recipients. Second, it was a single-centered study, and the findings may not be representative of other centers with different patient demographics, clinical practices, or healthcare settings. Third, the cross-sectional nature of the study does not provide information on the long-term outcomes of patients with CMV colitis or the efficacy of different management strategies over time. Fourth, the study did not analyze the impact of different immunosuppressive regimens in detail, which could be a significant factor influencing the prevalence and severity of CMV colitis in KTRs.

By acknowledging these limitations, future research can be designed to address these gaps, potentially involving larger, multi-center studies with diverse populations and a more comprehensive analysis of immunosuppressive therapies and long-term patient outcomes.

## Conclusions

This study investigated the prevalence and clinical implications of cytomegalovirus (CMV) colitis in renal transplant recipients (KTRs) presenting with lower gastrointestinal (GI) bleeding. The prevalence of CMV colitis in our population is comparable to some studies but lower than the others reported globally, suggesting geographic and methodological variations in CMV colitis diagnosis and prevalence.

Prophylaxis and early intervention are crucial in managing CMV infections. The study highlights that CMV prophylaxis, particularly during periods of high immunosuppression, can significantly reduce the incidence of CMV-related complications, thereby enhancing graft function and patient survival. These findings support the integration of routine CMV screening and prophylactic strategies into the post-transplant care protocols.

CMV colitis remains a significant concern for KTRs presenting with lower GI bleeding. Early detection and management through vigilant screening and prophylaxis can improve clinical outcomes. This study adds to the body of knowledge on CMV colitis in KTRs, underscoring the need for continued research and improved clinical practices to mitigate the impact of this condition on transplant recipients.
